# Two Approaches to the Laser-Induced Formation of Au/Ag Bimetallic Nanoparticles in Supercritical Carbon Dioxide

**DOI:** 10.3390/nano11061553

**Published:** 2021-06-11

**Authors:** Alexey Rybaltovsky, Evgeniy Epifanov, Dmitriy Khmelenin, Andrey Shubny, Yuriy Zavorotny, Vladimir Yusupov, Nikita Minaev

**Affiliations:** 1 Skobeltsyn Science Research Institute of Nuclear Physics, Lomonosov Moscow State University, 119991 Moscow, Russia; alex19422008@rambler.ru (A.R.); ummagumma44@yahoo.com (Y.Z.); 2 Institute of Photon Technologies, Federal Scientific Research Centre “Crystallography and Photonics” RAS, 108840 Moscow, Russia; rammic0192@gmail.com (E.E.); deerhunter9136@gmail.com (A.S.); iouss@yandex.ru (V.Y.); 3 FSRC “Crystallography and Photonics” RAS, 119333 Moscow, Russia; xorrunn@gmail.com

**Keywords:** laser ablation, supercritical fluid, supercritical carbon dioxide, plasmonic nanoparticles

## Abstract

Two approaches are proposed for the synthesis of bimetallic Au/Ag nanoparticles, using the pulsed laser ablation of a target consisting of gold and silver plates in a medium of supercritical carbon dioxide. The differences between the two approaches related to the field of “green chemistry” are in the use of different geometric configurations and different laser sources when carrying out the experiments. In the first configuration, the Ag and Au targets are placed side-by-side vertically on the side wall of a high-pressure reactor and the ablation of the target plates occurs alternately with a stationary “wide” horizontal beam with a laser pulse repetition rate of 50 Hz. In the second configuration, the targets are placed horizontally at the bottom of a reactor and the ablation of their parts is carried out by scanning from above with a vertical “narrow” laser beam with a pulse repetition rate of 60 kHz. The possibility of obtaining Ag/Au alloy nanoparticles is demonstrated using the first configuration, while the possibility of obtaining “core–shell” bimetallic Au/Ag nanoparticles with a gold core and a silver shell is demonstrated using the second configuration. A simple model is proposed to explain the obtained results.

## 1. Introduction

The recent increase in interest in the synthesis of bimetallic nanoparticles of silver and gold (Ag/Au BMNPs) is associated with their unique plasmonic and catalytic properties [1,2,3]. It is possible to purposefully change their characteristics and give them new functional properties by varying the elemental composition and morphology of such particles [4,5,6,7]. Due to this behavior, Ag/Au BMNPs can serve as the main elements in sensitive spectroscopic systems based on SERS, which can be used for detecting small amounts of bioorganic molecules or can be used in optical systems as detectors for recording changes in certain characteristics of liquid media, such as the refractive index [4,6].

One of the first methods for the synthesis of Ag/Au BMNPs was the chemical method, which is implemented through the preparation of a mixture of solutions of inorganic compounds of gold and silver, such as AgNO_3_ and HAuCl_4_ [8]. Recently, synthesis technologies based on the methods of “green chemistry” have been developed. These include methods using plant extracts as the biogenic agents, allowing Au NPs, Ag NPs, and Ag/Au BMNPs of various types to be obtained [3,9,10]; methods of biosynthesis of functionalized nanoparticles using biomolecules from microorganisms as the reactants, and capping agents of noble metals of microorganisms [11,12].

Simultaneously with the above approaches, methods based on laser ablation in various media have been developed. Their advantages include higher productivity and flexibility compared to other methods for synthesizing metal nanoparticles [5,13,14,15,16,17]. Currently, many methods for the synthesis of Ag/Au BMNPs using laser ablation are already known. For example, in some cases, the effect is achieved due to the action of laser radiation on thin films of silver and gold [14]. In other experiments, the required concentrations of gold and silver nanoparticles are first produced in separate volumes by laser ablation of the corresponding massive targets, and then the mixture of colloids is subjected to laser action [6]. Some articles [3,6,8,14] have noted the possibility of varying the molar composition of the obtained Ag/Au BMNPs by changing the content of the initial components for synthesis. In this way, it is possible to obtain not only nanoparticles with different plasmonic characteristics, but also with different morphologies—either the “alloy” type or the “core–shell” type [3].

The efficiency of the ablation process, the particle size distribution, their morphology, and other characteristics are influenced by the environment, the geometry of the experiment, and the parameters of the laser action (wavelength; energy, duration, and frequency of pulses; focusing parameters). In most works devoted to the formation of Au NPs, Ag NPs, and Ag/Au NPs, ablation was carried out in liquid, mainly in water [5,8,13,14,15]. Vacuum [18], gas [19,20], and supercritical carbon dioxide (scCO_2_) [7,21] have also been used as ablation media.

The use of a scCO_2_ medium for laser ablation is of interest because this medium has a number of unique properties that affect the formation, modification, and deposition of nanoparticles. The fundamental difference between a supercritical fluid medium and liquid media is the absence of an overheating regime and, as a consequence, the virtual absence of explosive boiling and the formation of vapor gas bubbles during pulsed laser heating. An important advantage of supercritical fluids is the ability to adjust the density and a number of other characteristics of the medium within a wide range by changing the temperature and pressure. This allows the ablation process to be controlled by selecting the optimal parameters of the medium, including during laser exposure [22]. The supercritical state of CO_2_ is characterized by extremely low viscosity at the level of gases, and high mobility of molecules, which allows ablation products to penetrate into hard-to-reach cavities and pores of various materials. At the same time, scCO_2_ is a strong nonpolar solvent and a cheap and environmentally friendly material, so it is widely used in the development of new technologies in the field of “green” chemistry [23,24,25]. Unlike organic media, scCO_2_ creates practically no byproducts arising from the photodegradation of organic molecules as a result of laser action.

This work is devoted to the development of approaches to the synthesis of Ag/Au BMNPs using the method of laser ablation of massive targets in supercritical fluid media. As in our first experiments on this topic [7,22], we used targets consisting of silver and gold plates. The scCO_2_ medium was chosen as the working medium in which the production of nanoparticles occurred.

We aimed to find specific experiment configurations to synthesize Ag/Au BMNPs by pulsed laser ablation of a target, which would make it possible to implement different morphologies of Ag/Au BMNPs. Two ablation modes were implemented with different sources of laser radiation and optics schemes, different geometrical configurations of the target, and different laser beam locations. In the first regime, at a peak power density of laser pulses of ~33 GW/cm^2^ and a large spot size on the target surface (Ø: ~0.3 mm), scanning by radiation over the target surface was not performed. In this case, the pulse repetition rate was low (50 Hz), and the laser radiation was supplied to the target horizontally. In the second regime using the same laser peak power density but a small spot size (Ø: ~45 μm), continuous scanning was carried out over the target surface. In this case, the pulse repetition rate was high (60 kHz), and the laser radiation was supplied to the target vertically.

## 2. Materials and Methods

**Figure 1:**
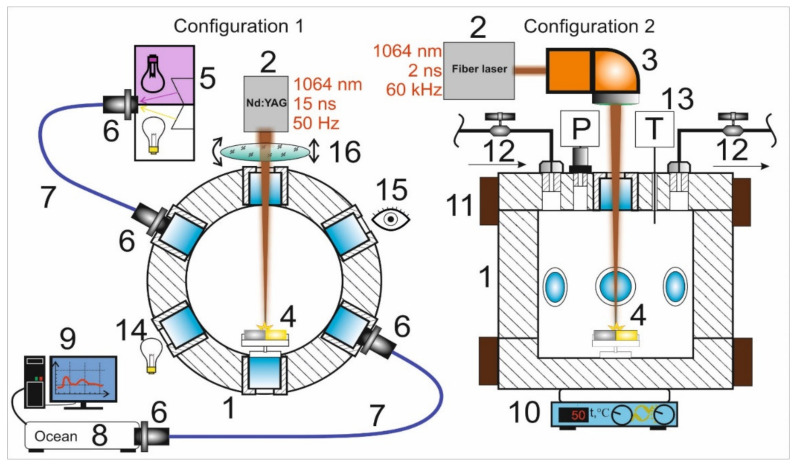
Schemes of installations for the synthesis of Ag/Au BMNPs show two different models of ablation. The schematic sections of the reactor, showing top (for configuration 1) and side views (for configuration 2): 1—high-pressure reactor; 2—laser sources; 3—galvo scanner with F-theta lens; 4—gold and silver targets; 5—UV and visible light sources; 6—collimator lens; 7—optical fibers; 8—spectrometer; 9—PC; 10—heating plate; 11—ring heaters; 12—needle valves for CO_2_ inlet and outlet; 13—pressure and temperature sensors; 14—backlight; 15—observation window; 16—quartz lens.

Experiments on the synthesis of Ag/Au BMNPs using the pulsed laser ablation of a double target were carried out in two configurations of the experimental setup (Figure 1), corresponding to two different ablation modes. The facilities were based on a high-pressure reactor (1) with a set of optical ports. The reactor had a modular design that made it possible to implement various configurations of laser action on the target with simultaneous diagnostics of the ongoing physical and chemical processes, using visual observation and spectral measurement methods. The reactor was equipped with six transparent 10-mm-thick quartz windows arranged in a hexagonal pattern in the horizontal plane, as well as an additional window in the upper cover of the reactor. Control over the process of laser ablation of targets in all experiments was carried out visually through the windows (15) using a digital camera. In the first configuration, the target was fixed on the side wall of the reactor, while in the second configuration, the target was mounted on the bottom of the reactor.

To achieve the first ablation regime (configuration 1), a Lotis LS-2138TF Nd:YAG laser (Minsk, Belarus) with an average power of P_L_ = 11 W operating in Q-switching mode was used as the source of laser radiation. Laser radiation (λ = 1064 nm, τ = 15 ns, E_p_ = 220 mJ, f = 50 Hz) was introduced into the internal volume of the high-pressure reactor through a side window and focused on the target using quartz lenses with a focal length of 8 cm (16). We used non-sharp focusing of the laser radiation with a spot size on the target surface of Ø 0.30 ± 0.02 mm. The target was gradually moving towards the focal plane of the laser beam along the path of the beam. In this case, with this spot size at the time of the onset of ablation, the optical breakdown of the medium occurred directly on the target surface. The peak power density of laser pulses on the target was 33 ± 5 GW/cm^2^.

To implement the second ablation regime (configuration 2), we used the radiation of a YLPP-1-150V-30 fiber laser (IPG Laser GmbH, Burbach, Germany) with an average power of P _L_= 30 W. Laser radiation (λ = 1064 nm, τ = 2 ns, E_p_ = 0.5 mJ, f = 60 kHz) was introduced into the reactor through the upper window. Focusing and movement of the laser beam were carried out using an LscanH-10 galvanic scanning system (Ateko-TM, Moscow, Russia) with an SL-1064-50-63 F-theta lens (Ronar-Smith, Singapore). The optical system made it possible to form a laser spot with Ø 45 ± 5 μm on the Au–Ag target. In the experiments, we used targets made of gold (99.95%) and silver (99.8%) with dimensions of 10 × 5 × 1 mm^3^, located side-by-side inside the reactor on a PTFE holder. The peak power density of the laser pulses in the focusing region, as in the first regime, was 34 ± 8 GW/cm^2^.

In the second regime (configuration 2), the laser radiation spot was moved using a galvanoscanner over the target surface at a speed of 100 mm/s, filling a rectangle of the selected size with a fill density of 100 lines per mm. The dimensions of these rectangles in the case of ablation of individual parts of the target made of gold or silver were 1 × 1 mm^2^, while in the case of simultaneous ablation of two parts of the target were 1 × 5 mm^2^.

To record the extinction spectra in both configurations, a fiber-optic spectroscopic system was assembled (5–9). The spectra were recorded using MAYA2000 PRO (spectral range: 200–1100 nm) and QE65000 (350–1100 nm) (Ocean Optics, Orlando, FL, USA) fiber spectrophotometers. Radiation from a halogen lamp and a deuterium gas discharge lamp (5) was introduced into the volume of the high-pressure reactor using 74UV collimators (Ocean Optics) (6) and an optical fiber (7). The radiation that passed through the reactor volume was collected by a collimator and entered the spectrometer (8). The spectra were recorded during the entire experiment with intervals ranging from 1 to 5 s.

The formation of Ag/Au BMNPs in both configurations consisted of successive laser action cycles on parts of the Au and Ag targets in scCO_2_ (P = 200 bar, T = 50 °C). In configuration 1 (Figure 1), a sequence of three ablation cycles of the golden part of the target was carried out as Au → Au → Au, then a sequence of three ablation cycles of the silver part of the target was carried as Ag → Ag → Ag. The duration of each cycle was 1 min, with 1 min intervals between cycles. In configuration 2 (Figure 1), one ablation cycle for the golden part of the target, one cycle for the silver part of the target, then one cycle simultaneously ablating both parts (three cycles in total) were carried out. The duration of each cycle was 10 min, with 20 min intervals between cycles.

The structure of the obtained nanoparticles was studied by transmission electron microscopy (TEM), scanning transmission electron microscopy with a wide-angle dark field detector (HAADF STEM), and energy-dispersive analysis (EDX) using FEI Osiris (FEI, Lincoln, NE, USA), FEI Scios (FEI), and Phenom PROX (Thermo Fisher, Eindhoven, Netherlands) electron microscopes. When using the TEM method, samples in the form of suspensions in organic solvents (after pretreatment in an ultrasonic bath for 15 min) were applied onto carbon-coated copper grids using the drop method. The collection of nanoparticles in the form of a suspension was carried out by washing them off the walls of the reactor with isopropyl alcohol. Measurements with an electron microscope were carried out on either the day of synthesis or the next day.

## 3. Results and Discussion

**Figure 2:**
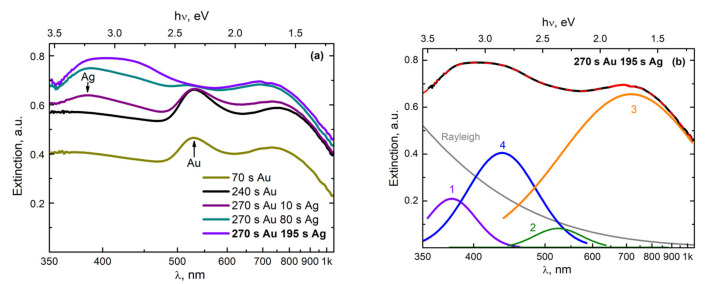
(**a**) Transformation of the extinction spectra of a colloidal solution of scCO_2_ during successive cycles of ablation of Au and Ag plates in configuration 1. The numbers corresponding to the curves show the times since the beginning of the ablation of the indicated (Au or Ag) target. (**b**) Variant of decomposition of the final spectrum (red line) into the sum (black dashed line) of individual Gaussian components (1–4) and the Rayleigh scattering component (Rayleigh).

The first results were obtained using a Lotis LS-2138TF laser source in configuration 1 (Figure 1), with a horizontal introduction of the beam onto the target. As a result of the laser action, the amplitude and shape of the extinction spectra gradually changed. The spectra were recorded continuously. Figure 2a shows the characteristic spectra obtained during the ablation of gold (times: 70 s and 240 s) and during the subsequent transition to the ablation of silver (times: 10 s, 80 s, and 195 s).

As a result of three successive gold target ablation cycles, an Au NP colloid was formed in the reactor. This led to the appearance of a plasmon resonance (PR) band with a maximum at the wavelength of 520 nm (Figure 2a) [22,26,27,28]. During the laser action for ~1 min (“70 s Au” curve, Figure 2a), the plasmon resonance band reached intensity saturation. At the same time, during the 1 min pause, the intensity hardly decreased, indicating low rates of aggregation and sedimentation processes of the Au NP colloid in scCO_2_. In the next stage, three consecutive cycles of laser ablation of a silver target were carried out, involving 1 min of ablation and a 1 min pause. This led to the efficient formation of a colloidal solution of plasmonic Ag NP. As a result, an increase in the absorption of the medium inside the reactor was observed and a PR band appeared on the extinction spectrum with a maximum at the wavelength of 380 nm (“240 s Au” curve, Figure 2a), corresponding to Ag NPs with a size of 4–10 nm [7,22,26,29]. However, it should be noted that during the pause, the intensity of this band decreased much faster than in the case of the colloid with Au NPs. We believe that this was due to the more efficient aggregation of small silver nanoparticles into large ones as compared to gold nanoparticles, followed by their gravitational sedimentation to the bottom of the reactor [22]. The faster aggregation of Ag NPs can be explained by the higher energy activation ability of silver atoms to form chemical compounds and complexes in comparison with gold atoms [28]. At the same time, it has been argued [3] that the gold atoms in liquids are able to participate more efficiently in comparison with silver atoms in the processes of their self-assembly into nanoparticles. It is likely that at the initial stage of the synthesis of such metal nanoparticles, this process is primarily influenced by the interactions of metal atoms with molecules from the environment.

Upon ablation of a silver target, simultaneously with an increase in the PR band with the maximum at 380 nm, a rapid degradation of the PR band with a maximum at 520 nm occurred, which was even previously retained during pauses. At the same time, a new band with a maximum at 420 nm appeared on the long-wavelength wing of the PR band, with a maximum at 380 nm. This effect was especially pronounced during pauses, when the intensity of the component from the PR of silver dropped and another component remained, which we believe was from Ag/Au BMNPs. In Figure 2b, the decomposition of the final spectrum into separate components can be seen. The experimental spectrum is shown with a black dashed line, while the sum of the five proposed components is shown with a solid red line. It was observed that in addition to the PR bands corresponding to Ag NPs (curve 1, maximum at 380 nm), there were Au NPs (curve 2, maximum at 550 nm) and possibly AuNP aggregates (curve 3, maximum at 750 nm); curve 4 (maximum at 420 nm) can be attributed to Ag/Au BMNPs. Regarding differences in the positions of the maximum and the widths of the PR band for single Au NPs obtained in this expansion in comparison with the results in Figure 2a, these can most likely be associated with the manifestation of the PR band from some residual Au NP fraction (larger), which had not yet entered the “hot zone” near the silver target.

**Figure 3:**
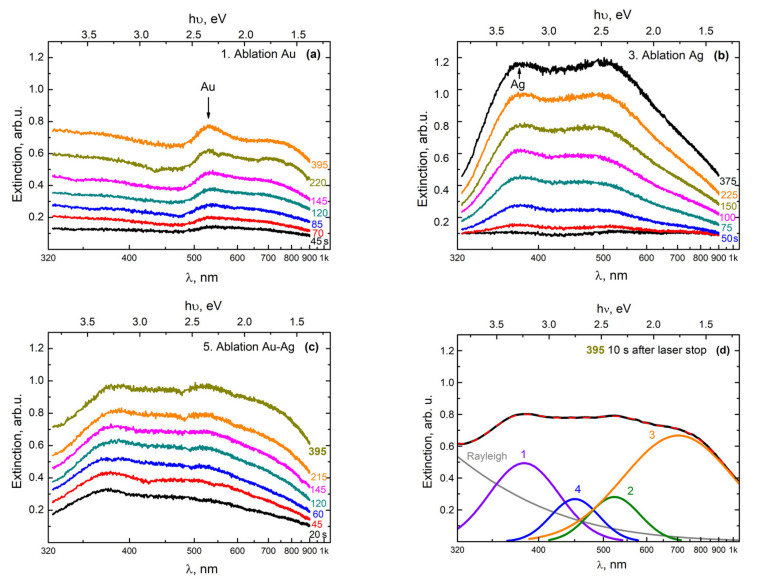
Transformation of the extinction spectra of a colloidal solution scCO_2_ during successive cycles of ablation of the (**a**) gold and (**b**) the silver parts of the target. (**c**) Simultaneous ablation of both parts in configuration 2. The numbers on the curves show the times from the beginning of the ablation in seconds. The numbers to the right of the curves show the times in seconds since the start of the ablation cycle. (**d**) Variation of the decomposition of the spectrum obtained 10 s after the end of the last stage of ablation (red line) into the sum (black dashed line) of individual Gaussian components (1–4) and the Rayleigh scattering component (Rayleigh).

A similar transformation of the extinction spectra was observed (Figure 3) when using a YLM-1-150V-30 laser source in configuration 2 (Figure 1), with a vertical insertion of the beam onto a double target of gold and silver located at the bottom of the high-pressure reactor.

In the first stage, an Au NP colloid was formed during the ablation of a gold target, as in the previous case. This led to the appearance in the extinction spectrum of the colloid of the PR band, with the maximum at 520 nm (Figure 3a). In the next stage of ablation of the silver target, a broad band appeared with a maximum at 380 nm (Figure 3b), which corresponded to the PR of Ag NPs with a size of 4–10 nm [22]. Note that simultaneously to the growth of this maximum during the second stage, the amplitude of the maximum corresponding to the PR from gold nanoparticles also increased.

We believe that this effect was due to the fact that at the location of the target at the bottom of the reactor, the large gold nanoparticles that formed in the colloid during the first stage of ablation were also effectively deposited onto the surface of the silver target. Therefore, in the second stage (during the ablation of the silver target), the silver target was actually ablated together with the surface layer consisting of Au NPs, which were deposited onto the surface as a result of the ablation of the gold target. The presence of this deposited Au NP film on the target plates was confirmed by visual observations when the reactor was opened after the first cycle of the experiment. Figure 3c presents the extinction spectra, which were obtained in the process of ablation of gold and silver targets simultaneously, when scanning a laser beam over the surfaces of the two parts (Au and Ag) of the target. With a gradual increase in extinction in the entire observed wavelength range, the shape of the curves underwent transformation, in which an absorption band corresponding to Ag/Au BMNPs appeared against the background of PR bands from Ag NPs and Au NPs, as well as a long-wavelength absorption band.

Figure 3d shows a variation of the decomposition of the extinction spectrum of a colloid, obtained as a result of the simultaneous ablation of two parts of the target (Au and Ag) in configuration 2. It was observed that in addition to the PR bands of silver nanoparticles (curve 1, the peak with the maximum at 380 nm) and gold (curve 2, the peak with the maximum at 520 nm), there were also long-wave absorption bands (curve 3, the peak with the maximum at 700 nm), which can be classified as large nanoparticles and their aggregates. Additionally, in Figure 3d, band 4 (the peak with the maximum at 450 nm) stands out; in our opinion, it comprises Ag/Au BMNPs, but most likely is of a slightly different type, meaning it differs from the particles obtained in configuration 1.

The large width of the obtained spectra is associated with a rather strong scattering of NPs in terms of the composition (Ag/Au ratio), shape, and size. However, the obtained results can be considered preliminary, which makes it possible to reveal the specifics of the experimental parameters and the regularities for the preferential synthesis of bimetallic NPs of a certain type.

**Figure 4:**
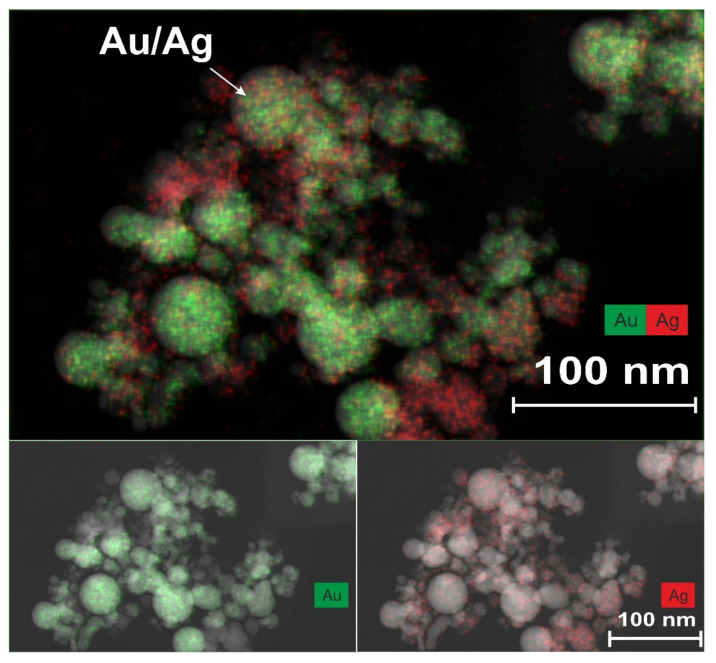
TEM images of nanoparticles obtained using the EDX method. The arrow indicates one of the largest Ag/Au-BMNP-type “alloys”, measuring up to 40 nm in size. Nanoparticles were synthesized in the setup of configuration 1, using a low-frequency laser source with a high pulse energy.

The TEM analysis of the obtained nanoparticles using the EDX method turned out to be informative and confirmed the stated provisions on the formation of bimetallic nanoparticles of various types. A similar method using the EDX system for the analysis of the compositions of composite nanoparticles was successfully used by the authors of another study [30] in the synthesis of carbon-coated Au NPs in the process of pulsed laser ablation of a gold target in a scCO_2_ medium. Figure 4 shows a TEM image of nanoparticles, which was obtained using the EDX method during laser ablation of a double target in configuration 1.

In the upper region of this figure, nanoparticles measuring less than 10–20 nm can be observed, the PR bands of which are in the visible and near-UV wavelength ranges. Indeed, the maxima of the PR bands shown in Figure 2 for NP are in the range of 380–520 nm. The band with a maximum at 380 nm corresponds to small silver nanoparticles in scCO_2_. Usually, for such particles dispersed in organic solvents, solid matrices, or water, the maximum of the PR band is observed in the range of 410–420 nm [26,28,29,31], and the short-wavelength shift for such a PR arises from other dielectric properties of the scCO_2_ medium. It is likely that a certain shift in the PR band for small Au NPs will also occur when they are placed in a supercritical medium. In our situation, the maximum of the PR band for such particles based on Figure 2 is in the region of 520 nm, while for similar particles in organic or aqueous media, the maximum is at 540 nm [27,31]. According to [3], with a homogeneous distribution of Au and Au atoms during the formation of bimetallics of the “alloy” type, the maximum of the PR band smoothly shifts to the short-wavelength region from the position at 540 nm (at 100 mol % Au), with an increase in the molar content of the silver atoms contained within. The appearance in the PR spectrum of a component with a maximum at 420 nm (see Figure 2b) may well correspond to the synthesis of bimetallic nanoparticles of the “alloy” type, whereby the content of silver atoms, taking into account the above considerations, should be in the range of 60–80 mol %. The observed color gamut, which corresponds to the mixed elemental composition of the Ag/Au BMNPs, one part of which is indicated by an arrow, also does not contradict the reasoning given here.

**Figure 5:**
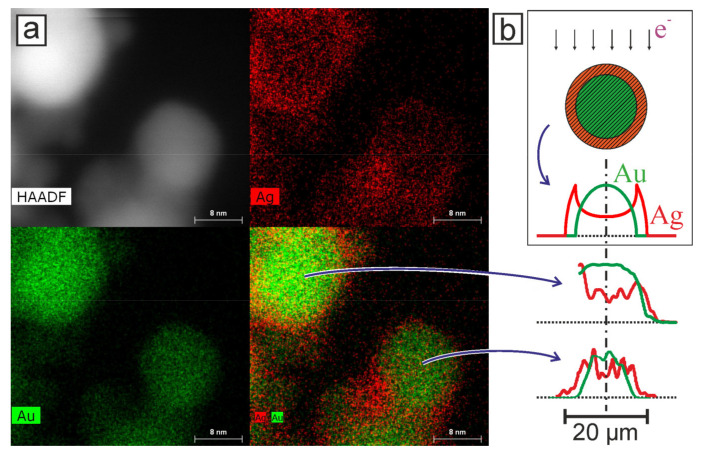
(**a**) TEM images of Au/Ag “core–shell” BMNPs, demonstrated using the EDX technique. Nanoparticles were obtained by laser synthesis in configuration N2. (**b**) Top: A cross-sectional image of a nanoparticle model with a core–shell structure and the pixel intensity profile of the elements from the core and shell for the corresponding EDX TEM image. Arrows show the direction of the flow of electrons (e-) during image acquisition. Bottom: Pixel intensity profiles for Au and Ag for TEM images of two nanoparticles.

Figure 5 shows TEM images of nanoparticles that appeared during the ablation of a double target in configuration 2. In this figure, using the EDX technique, two particles can be distinguished, which can be attributed to the bimetallics. Figure 5b shows the profiles of the intensity distribution of pixels corresponding to Ag and Au elements from two nanoparticles from Figure 5a. Comparison with the corresponding distribution for a model nanoparticle with a core–shell structure (Figure 5b, top) shows that the two isolated nanoparticles also have a core–shell structure. It can be observed that these Ag/Au BMNPs are composed of a gold core and a silver shell. The core and cladding diameters are 17 ± 1 and 19 ± 1 nm for the larger Au/Ag BMNPs and 13 ± 1 and 15 ± 1 nm for the smaller ones, respectively.

In these experimental studies, we used two configurations of installations (Figure 1), allowing two different ablation regimes at the same peak power densities (~33 GW/cm^2^).

**Figure 6:**
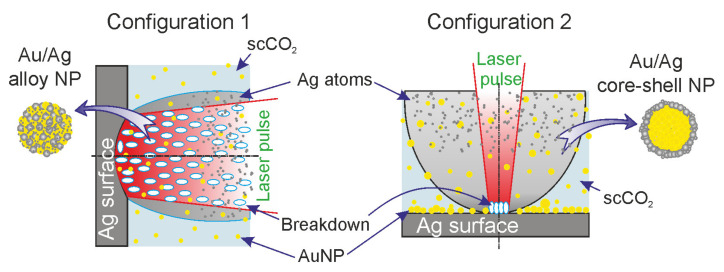
Model representation of the processes occurring during the ablation of a silver target in two different configuration setups (Figure 1) and leading to the synthesis of Au/Ag BMNP “alloy” (configuration 1) and “core–shell” (configuration 2) types.

In the first configuration (Figure 1), laser action with a low pulse repetition rate (f = 50 Hz) was carried out horizontally without moving the laser spot over the target surface. As a result of the ablation, a crater with a diameter of 300 μm formed on the target. When implementing this regime, it was planned that the synthesis of Ag/Au BMNPs would mainly occur during the action of a laser pulse in a sufficiently wide laser beam (Figure 6). In the case of large laser spots, the ejection of the silver target material occurred mainly perpendicular to its surface towards the beam. As shown in [32], with increases in the size of the plasma source over 20 μm, its density on the optical axis gradually increased. The formation of a flux of atoms, ions, and electrons directed perpendicular to the target surface was also facilitated by the formation of a crater on the surface of the silver target. Such a directed flux of particles from the target led to numerous optical breakdowns in the region of the laser beam (Figure 6). A visual confirmation of this effect was the observed bright laser track in the scCO_2_ medium in the first configuration of the experiment. Such a breakdown was accompanied by a sharp increase in temperature throughout the entire region and the formation of an ion atom plasma with Ag and Au. Further self-assembly of these particles led to the predominant formation of Au/Ag BMNPs of the “alloy” type, which were recorded using TEM (see Figure 4).

In the second configuration (Figure 1 and Figure 6), laser action with a pulse frequency of 60 kHz was carried out vertically. In this case, the spot size on the target surface was relatively small (Ø: 20 μm) and constantly moved over the target surface. With this configuration, during the ablation of the gold target (at the first stage), a layer of Au NPs was deposited on the surface of the silver part of the docked target due to the effect of gravitational sedimentation. In this case, with further ablation of the silver target, the removed target material interacted not only with the colloid but also with the gold nanoparticles on its surface. It should also be taken into account that due to the small size of the laser spot on the target and the absence of a crater comparable in area to the spot size, the substance in this case was carried into the colloid at a wide solid angle [32]; thus, when this configuration was implemented, the synthesis of Ag/Au BMNPs mainly occurred not in a relatively narrow laser beam but in a sufficiently large volume of the colloid into which the target material was ejected (Figure 6). In this volume, the deposition of Ag ions and atoms on the surfaces of Au NPs contained in scCO_2_ took place with the formation of Au/Ag BMNPs of the “core–shell” type (see Figure 5). It is noted that in this configuration, due to the wide scattering angle of the silver target material, breakdown events occurred only in the immediate vicinity of the target surface at a relatively small volume, as was observed visually during the experiment. Therefore, the probability of the formation of Au/Ag BMNPs of the “alloy” type in configuration 2 was several orders of magnitude lower than in configuration 1.

The mechanisms of the formation of small bimetallic nanoparticles in the scCO_2_ colloid in the two presented configurations were different. These different mechanisms led to the predominant formation of Au/Ag BMNPs of the “alloy” type (Figure 4) in configuration 1 and Au/Ag BMNPs of the “core–shell” type in configuration 2.

In configuration 1 (Figure 6), Au/Ag BMNPs of the “alloy” type (Figure 4) were synthesized in scCO_2_ from a cloud of Ag and Au ions and atoms in a wide laser beam. The formation of this cloud occurred as a result of numerous breakdown events caused by the interaction of a laser pulse with a strongly directed flux of particles from an Ag plate flying towards it. In configuration 2 (Figure 6), Au/Ag BMNPs of the “core–shell” type (Figure 5) were synthesized, mostly from a cloud of Ag ions and atoms and Au NPs in a wide region formed by a weakly directed flow of particles from an Ag plate [33,34,35].

In general, the formation of bimetallic nanoparticles in scCO2 occurred as a result of (1) the self-assembly of atoms, (2) the aggregation (or coalescence) of nanoparticles and atoms, or (3) autocatalytic growth [36]. Some of these structures in the process of enlargement can form Ag or Au nanoparticles. The others can be transformed into bimetallic nanoparticles of the “core–shell” type [3,4,6,35] (Figure 5) or form nanoparticles of the “alloy” type mixed from Au and Ag atoms (Figure 4). It should be noted that the proposed simple model shows possible ways of obtaining NPs with different Ag/Au ratios. For example, if it is necessary to obtain gold nanoparticles of the core–shell type with a thinner silver layer on the surface of the gold core, configuration 2 will reduce the laser intensity or the silver ablation time.

Due to the vortex and convection flows arising from pulsed laser heating, the formed nanoparticles are scattered throughout the reactor volume and enter the observation zone, causing the appearance of characteristic plasmon resonance absorption bands. As noted in our first experiment [7], as well as in other papers on this topic [4,5,33], the PR bands for Au/Ag BMNPs are located in the interval between the known PR bands of pure silver and gold. Our explanation for the mechanism of the predominant formation of bimetallic nanoparticles of different types in two configurations does not contradict the considerations expressed in a previous [3], where the authors argue that the formation of Ag/Au BMNPs of one type or another largely depends on the ratio of silver and gold atoms in the reaction zone; with a significant excess of gold atoms in a liquid medium, these primarily combine with each other due to higher rates of movement in this environment in comparison with silver atoms. Indeed, in our situation, in the presence of an Au NP layer formed on the surface of a silver target in a scCO_2_ medium, this phenomenon is possible.

It is important to note that the proposed specific approaches to the implementation of Au/Ag BMNP synthesis using a supercritical fluid medium belong to the field of “green chemistry”. First of all, scCO2 is usually used as a substitute for organic solvents, which allows one to get rid of large volumes of liquid waste. In addition, scCO_2_ can be converted into a gaseous state during the synthesis process, which makes it possible to implement a closed production cycle without the emission of pollutants.

## 4. Conclusions

Two different modes of laser action on a target consisting of two plates of Au and Ag in supercritical carbon dioxide in two different configurations were considered. The configuration for the experiment and the mode of laser action—with the same peak power density for the laser pulses (~33 GW/cm^2^)—played fundamentally important roles in the production of bimetallic Au/Ag nanoparticles of both the “alloy” and “core–shell” types (with a gold core and silver cladding).

In configuration 1, when the double target was located vertically on the side wall of the chamber and its components were ablated alternately by a stationary wide beam of a low-frequency laser with a high pulse energy, Au/Ag BMNPs of the “alloy” type were predominantly formed. In the case of configuration 2, in which the double target was located horizontally at the bottom of the chamber and the ablation of its components occurred alternately by scanning a narrow beam of a high-frequency laser, Au/Ag BMNPs of the “core–shell” type were predominantly formed. A simple model was proposed that explains the predominant formation of the two types of nanoparticles (core–shell type or alloy type) with different experiment configurations and laser action parameters.

## Data Availability

The data presented in this study are available on request from the corresponding author.
